# GDF15: A Novel Insight Into Its Biology and Therapeutic Potential in Obesity

**DOI:** 10.1155/jdr/3024772

**Published:** 2026-07-22

**Authors:** Kamil Ahmed, Asia Saturnino, Paolo Pozzilli

**Affiliations:** ^1^ Queen Mary University of London, London, UK, qmul.ac.uk; ^2^ Department of Endocrinology and Diabetes, Fondazione Policlinico Universitario Campus Bio-Medico, Rome, Italy; ^3^ The Blizard Institute, Queen Mary University of London, London, UK, qmul.ac.uk

**Keywords:** body weight, cellular stress signalling, growth differentiation factor 15 (GDF15), obesity, therapy

## Abstract

Growth differentiation factor 15 (GDF15) is a stress‐responsive cytokine that has recently been identified as a key regulator of appetite homeostasis. Although GDF15 was previously regarded as only a biomarker of disease burden, the discovery of its exclusive receptor, GFRAL, repositioned it as a key mediator of sickness‐associated anorexia. The clinical significance of GDF15 and its receptor, GFRAL, is supported by evidence that established weight‐loss agents influence circulating GDF15 levels and that GDF15 agonists induce marked weight loss in both preclinical and early‐phase clinical studies. This review integrates recent advances in GDF15 biology from evolutionary, mechanistic and translational perspectives, with particular emphasis on its emerging therapeutic potential in obesity.

## 1. Introduction

Obesity is a growing global public health crisis [1], with over 890 million adults affected worldwide in 2022. It is recognised as a systemic disorder with far‐reaching effects beyond impaired glucose metabolism, including chronic inflammation and maladaptive stress responses [2].

Pharmacotherapies such as GLP‐1 receptor agonists have demonstrated substantial clinical efficacy, yet treatment response remains heterogeneous, and durable weight loss is not achieved in all individuals.

Data from the STEP 2 trial in type 2 diabetes indicate that semaglutide 2.4 mg produced clinically meaningful weight loss, although approximately 31.2% of participants did not achieve a 5% weight reduction [3]. These data support the effectiveness of these therapies while also highlighting the need to better understand complementary pathways regulating food intake and energy balance. Among these, the GDF15–GFRAL axis has emerged as a distinct stress‐responsive pathway linking peripheral cellular distress to appetite suppression [4]. However, despite interest in GDF15 as a biomarker and possible therapeutic target, it remains severely underexplored.

**Figure 1:**
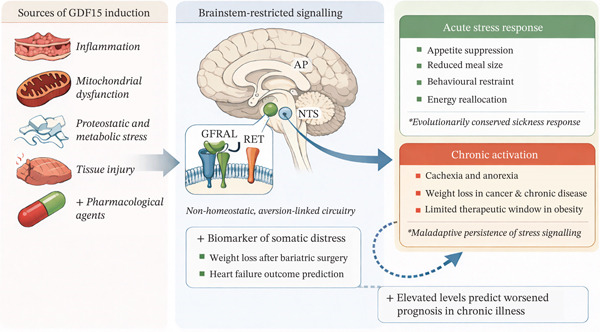
The spectrum of biological actions of GDF15 across physiological and pathological contexts. GDF15 is a stress‐responsive cytokine induced by diverse stimuli, including mitochondrial dysfunction, inflammation and pharmacological agents such as metformin. The protein acts as an endocrine signal that binds exclusively to the GFRAL–RET receptor complex, which is anatomically restricted to the area postrema (AP) and nucleus tractus solitarius (NTS) of the hindbrain. Acute activation triggers an evolutionarily conserved sickness response characterised by appetite suppression and behavioural restraint. In contrast, chronic elevation is a marker of systemic physiological burden, contributing to maladaptive outcomes such as cancer cachexia, anorexia and frailty in chronic disease states.

This review is aimed at addressing these unexplored aspects by integrating evidence from evolutionary biology, cellular stress signalling and translational medicine to evaluate the dual role of GDF15 as both a biomarker and effector of systemic metabolic stress, and at assessing its emerging therapeutic potential in obesity (Figure 1).

## 2. Evolutionary Biology

GDF15, originally identified as macrophage inhibitory cytokine‐1 (MIC‐1), is a cysteine‐knot protein belonging to the transforming growth factor‐*β* superfamily that is upregulated in response to cellular stress [5]. For many years, it was regarded as a nonspecific biomarker due to its broad elevation across various pathological states, including cancer and cardiovascular disease. This perspective shifted with the discovery of the glial‐cell–derived neurotrophic factor (GDNF) receptor alpha‐like (GFRAL) [6, 7].

GFRAL is expressed exclusively in the area postrema (AP) and nucleus tractus solitarius (NTS) in the hindbrain, which are regions associated with sickness behaviour and appetite suppression. This receptor distribution provided the first clear mechanistic explanation for GDF15′s anorectic effects, reframing GDF15 as not only a biomarker but also a hormone acting through a dedicated neuroendocrine axis, the GDF15–GFRAL axis [8].

The evolutionary significance of this pathway is demonstrated by the conservation in the mature GDF15 domain among mammalian species, especially in regions essential for GFRAL binding [6]. In contrast, GFRAL is absent in lower vertebrates such as amphibians and fish, suggesting that the GDF15–GFRAL axis evolved later in vertebrate evolution. This pattern suggests selective pressure to maintain a stress‐responsive anorectic signal that can suppress feeding during periods of physiological threat [8, 9].

In the context of somatic distress, elevated GDF15 levels suppress food intake and reduce locomotor activity. These responses are consistent with sickness behaviour across species [10]. From an evolutionary perspective, this transient anorexia likely provided adaptive benefits by limiting energy expenditure, reducing exposure to pathogens or toxins and reallocating resources to immune activation and tissue repair [11].

This interpretation is consistent with the broader concept of sickness anorexia, where inflammatory or toxic insults suppress feeding regardless of energy status [12]. The GDF15–GFRAL axis links peripheral stress signals to a brainstem‐restricted receptor system, enabling suppression of appetite when physiological risk is elevated. This adaptive framework also highlights an evolutionary trade‐off. In contemporary settings marked by chronic, low‐grade metabolic stress, ongoing activation of the GDF15 pathway may be maladaptive [13]. Sustained increases in circulating GDF15 are seen in conditions such as cancer, chronic kidney disease and age‐related sarcopenia, where prolonged anorectic signalling contributes to cachexia, frailty and functional decline [14, 15]. While GDF15 signalling is protective during acute stress, its chronic activation demonstrates the limitations of an evolutionarily conserved system functioning outside its original context.

## 3. Cellular Regulation of GDF15

At a transcriptional level, GDF15 induction is most consistently associated with activation of the integrated stress response (ISR). This response is activated when cells experience disruptions in proteostasis or bioenergetic capacity, leading to the phosphorylation of eIF2*α* and the downstream activation of stress‐responsive transcription factors such as ATF4 and CHOP [16–18]. The ISR represents a shared downstream pathway through which multiple cellular stressors converge, allowing selective translation of stress‐adaptive transcripts (notably ATF4) while reducing global protein synthesis [16–18]. Within this framework, GDF15 functions as a secreted readout of intracellular strain, allowing intracellular metabolic stress to be reflected in circulating hormone levels.

Distinct stressors activate different eIF2*α* kinases: Endoplasmic reticulum (ER) stress engages PERK, amino‐acid limitation engages GCN2, viral or inflammatory double‐stranded RNA signals can engage PKR and haem/oxidative stress can engage HRI. Despite these distinct origins, these pathways all converge on eIF2*α* phosphorylation and ATF4 induction [16–18].

Mitochondrial stress is a particularly relevant ISR trigger in metabolic disease, because impaired oxidative phosphorylation can generate reactive oxygen species (ROS), perturb NADH/NAD^+^ balance and reduce ATP availability—each of which increases proteostatic pressure and promotes ISR activation [10, 16–18]. Importantly, these stress pathways are not isolated. Mitochondrial dysfunction can amplify inflammatory cascades, whereas ER stress can alter lipid handling and cytokine production, compounding cellular strain. As a result, GDF15 induction often reflects the cumulative burden of stress rather than engagement of a single upstream pathway [10].

Multiple tissues contribute to circulating GDF15, including the liver, adipose tissue, skeletal muscle and immune cell compartments (Table 1). The relative contribution of each source appears to be context dependent. Acute inflammatory states may favour hepatic and myeloid production, whereas chronic metabolic disease likely reflects a composite signal arising from adipose inflammation, hepatic lipid burden and skeletal muscle bioenergetic stress [19]. This tissue nonreinforces the view of GDF15 as a nonspecific systemic marker of cellular stress rather than an organ‐specific hormone, and it also helps explain why similar body mass index (BMI) can mask very different biological stress loads across individuals.

**Table 1:** Context and physiological stimuli for GDF15 induction. This table summarises the primary tissue sources and the biological mechanisms driving GDF15 expression in response to cellular stress.

Context/stimulus	Primary tissue sources	Evidence for GDF15 induction	Key references
Cellular stress	Multiple tissues	Induced as a stress‐responsive cytokine across diverse pathological states, reflecting intracellular strain.	[4, 8]
Integrated stress response (ISR)	Liver, skeletal muscle, gut	Activation of ISR pathways (ATF4, CHOP) links proteostatic and metabolic stress to GDF15 expression.	[14, 15]
Mitochondrial dysfunction	Skeletal muscle, high‐demand tissues	Released as a ‘mitokine’ in response to impaired oxidative phosphorylation and bioenergetic stress.	[10, 13]
Inflammation and tissue injury	Immune and parenchymal cells	Inflammatory cytokines (e.g. IL‐1*β*) induce GDF15 to contribute to systemic tissue tolerance.	[9]
Chronic disease states	Tumours, myocardium, systemic tissues	Sustained elevation correlates with disease severity, cachexia and adverse outcomes.	[11, 18]
Bariatric surgery	Gut and systemic tissues	Postoperative rise in GDF15 correlates with total weight loss at 12 months.	[8, 31]

Abbreviations: ATF4, activating transcription factor 4; CHOP, C/EBP homologous protein; IL‐1*β*, interleukin‐1 beta; ISR, integrated stress response.

Regulation also occurs beyond transcription. Circulating GDF15 concentrations depend on secretion dynamics, the total mass of stressed tissue and renal function, with higher levels typically reported in chronic kidney disease [20]. Consequently, absolute values must be interpreted cautiously, especially in populations with impaired kidney function or multisystem disease. Serial measurements may therefore be more informative than single time‐point assessments when GDF15 is used to track disease trajectory or treatment response [21].

Finally, the stress‐linked nature of GDF15 distinguishes it from classical metabolic hormones. Whereas leptin broadly reflects fat mass and insulin reflects glycaemic status, GDF15 more closely mirrors systemic strain—encompassing inflammatory tone, proteostatic capacity and mitochondrial function [22]. This distinction provides a mechanistic basis for why GDF15 rises in conditions as diverse as obesity, malignancy, pregnancy and ageing, and why its elevation cannot be attributed solely to adiposity.

## 4. Cellular Signalling and Receptor Architecture

A defining feature of GDF15 signalling is that its biological effects are constrained not only by receptor localisation but also by the structural organisation of the signalling complex itself. Unlike classical metabolic hormones that act through autonomous receptors with intrinsic signalling capacity, GDF15 engages an obligate tripartite system composed of the ligand‐binding receptor GFRAL and the receptor tyrosine kinase RET [6, 7]. GFRAL functions as an orphan receptor lacking intracellular signalling domains, such that effective pathway activation depends entirely on RET recruitment and dimerisation. This architectural dependency imposes a high threshold for signalling and contributes to the extreme anatomical restriction of GDF15 responsiveness to discrete brainstem nuclei in which both components are coexpressed.

At the ligand level, mature GDF15 is secreted as a disulphide‐linked homodimer adopting a cysteine‐knot fold characteristic of the transforming growth factor‐*β* superfamily [5]. Despite this conserved structural motif, GDF15 exhibits remarkable receptor specificity, binding exclusively to GFRAL and not to other members of the GDNF receptor family [8, 9]. This molecular exclusivity insulates GDF15 signalling from parallel neurotrophic pathways and reinforces its role as a dedicated stress‐responsive axis rather than a broadly homeostatic regulator of energy balance.

Ligand binding drives assembly of a higher order signalling complex in which two GDF15 dimers engage two GFRAL molecules, permitting recruitment and autophosphorylation of RET [23, 24]. Since GFRAL lacks independent signalling capacity, pathway activation is effectively binary: Once RET is engaged, downstream signalling proceeds robustly rather than in a graded manner. This structural logic provides a mechanistic explanation for the steep dose–response relationships observed in pharmacological studies, where small increases in ligand exposure can rapidly transition from appetite suppression to the induction of aversive responses [25, 26].

Activated RET initiates canonical intracellular cascades, including phosphorylation of ERK1/2 and activation of phospholipase C‐*γ* (PLC*γ*) [23, 24]. These pathways modulate neuronal excitability and transcriptional programmes within the AP and NTS, enabling circulating stress signals to exert rapid central effects. The requirement for coordinated receptor assembly and coreceptor recruitment, therefore, links molecular structure directly to physiological outcome, positioning receptor architecture as a primary determinant of signalling behaviour.

Importantly, the obligate nature of RET recruitment also constrains signalling to a discrete neuronal population within the dorsal hindbrain. Coexpression of GFRAL and RET is largely restricted to the AP and adjacent NTS regions situated outside the conventional blood–brain barrier and specialised for chemosensory detection of circulating cues. This spatial confinement prevents engagement of broader hypothalamic energy‐balance networks and distinguishes GDF15 signalling from classical metabolic hormones that act through widely distributed receptors.

At the circuit level, activation of GFRAL–RET–expressing neurons preferentially engages projections to the parabrachial nucleus, a hub integrating visceral sensory input with autonomic and aversive responses. Structural coupling of ligand binding to robust RET activation therefore translates into behavioural restraint rather than graded satiety. Functionally, this organisation manifests as reduced meal size, conditioned avoidance and altered autonomic tone, while leaving mesolimbic reward circuitry relatively unengaged.

From a translational perspective, the same receptor architecture that confers specificity also imposes limitations. Binary activation of a brainstem circuit closely linked to aversion yields a narrow therapeutic window in which efficacy and tolerability remain tightly coupled. Unlike incretin‐based therapies, where downstream signalling can be partially dissociated from gastrointestinal side effects over time, the GDF15–GFRAL axis offers limited scope for adaptive separation of benefit and adverse response. Consequently, receptor structure itself emerges as a central constraint on the clinical viability of GDF15‐based interventions.

In summary, GDF15 signalling is defined by an obligate receptor architecture that enforces anatomical confinement, binary intracellular activation and engagement of aversion‐linked brainstem circuits (Table 2). This integration of molecular structure, cellular signalling and neural circuitry accounts for both the biological potency of GDF15 and the translational challenges that have thus far limited its therapeutic development.

**Table 2:** Biological features of the GDF15–GFRAL signalling axis. A summary of the structural and anatomical constraints that define the central mechanism of action for GDF15.

Domain	Key feature	Implication	Key references
Receptor localisation	GFRAL expression is restricted to the area postrema (AP) and nucleus tractus solitarius (NTS).	Enforces nonhomeostatic control of feeding via regions outside the blood–brain barrier.	[5, 6]
Receptor architecture	Obligatory GFRAL–RET complex formation.	Activates downstream ERK1/2 and PLC*γ* pathways to modulate synaptic plasticity.	[20, 21]
Neural circuitry	Engagement of aversion‐linked brainstem pathways projecting to the parabrachial nucleus.	Appetite suppression is tightly coupled to malaise and conditioned taste aversion.	[12, 22]
Systemic effects	Predominantly indirect peripheral actions.	Weight loss reflects central integration of stress signals rather than direct thermogenesis.	[8, 19]

Abbreviations: AP, area postrema; ERK1/2, extracellular signal‐regulated kinase; GFRAL, GDNF family receptor alpha like; NTS, nucleus tractus solitarius; PLC*γ*, phospholipase C‐gamma; RET, rearranged during transfection.

## 5. Therapeutic Development and Clinical Translation

The translation of GDF15 biology into viable therapeutics has required solutions to two linked constraints: (i) Native GDF15 is not an attractive chronic medicine because short half‐life and exposure variability make sustained dosing impractical, and (ii) even when exposure is engineered, the clinically usable dose is limited by the pathway′s tight coupling of anorectic efficacy to nausea and malaise [5, 23–28]. As a result, drug development has centred on long‐acting agonist formats to improve pharmacokinetics, alongside strategies intended to widen the practical therapeutic window.

Most programmes have used established protein‐engineering approaches—Fc fusion, PEGylation and stabilising substitutions—to prolong half‐life and support infrequent dosing [5, 22]. These modifications can improve systemic exposure and reduce dosing burden, but they do not inherently change what ultimately limits dose in humans: tolerability. In practice, pharmacokinetic optimisation may make weekly (or less frequent) dosing feasible, yet average weight loss depends on whether the required target engagement can be achieved below the nausea threshold [25–28].

Early‐phase trials with long‐acting GDF15 agonists have provided proof‐of‐mechanism in humans. LY3463251 (Eli Lilly) demonstrated appetite/food‐intake suppression with modest reductions in body weight over short treatment periods [26]. The key translational lesson was not a lack of biological activity, but that the dose range producing meaningful appetite suppression appears to overlap with the dose range producing aversive symptoms in a substantial proportion of participants [25–28]. This aligns with the underlying neurobiology: The therapeutic effect is mediated by circuits where anorexia and visceral malaise are closely linked outputs rather than separable endpoints [25–27].

A consistent feature of the pipeline has been discontinuation after disappointing mid‐stage efficacy despite strong preclinical effects. MBL949 is frequently cited as a clear example; while preclinical studies suggested robust weight reduction, human trials reported minimal weight loss, contributing to discontinuation [29]. Similarly, AMG 171 (Amgen) did not progress following early clinical evaluation [30]. Collectively, these outcomes illustrate a practical translation gap: Potent primate efficacy does not guarantee clinically meaningful human weight loss if tolerability caps achievable exposure or if compensatory responses blunt durability at the doses patients can remain on [5, 25–30].

Across programmes, the dominant barrier has been the narrow therapeutic index. In contrast to incretin‐based therapies—where gastrointestinal symptoms may attenuate in some patients with gradual titration—GDF15 agonism recruits circuits inherently linked to aversion, such that symptom burden is often mechanistically aligned with the anorectic effect [24–26]. Consequently, tolerability becomes the primary determinant of the maximum usable dose, which then caps achievable average population‐level weight loss even when biological potency is strong [25–28].

A promising strategy is to reduce reliance on high‐intensity GDF15 signalling by pairing it with complementary satiety pathways. QL1005, a GLP‐1/GDF15 dual agonist, exemplifies this approach by combining incretin‐mediated satiety with GDF15‐driven intake suppression [31]. In preclinical nonhuman primate studies, dual constructs have been reported to achieve ~10%–15% weight loss—greater than typical GDF15 monotherapy benchmarks—raising the possibility that efficacy can be achieved with a lower effective ‘GDF15 intensity’ and therefore improved tolerability through dose‐sparing [31]. Whether this advantage translates in humans remains uncertain: Dual agonism could still be limited by the aversive liability of the GDF15 component at clinically effective doses, and careful early‐phase work will be needed to demonstrate that symptom burden does not scale in parallel with efficacy [27, 28, 31].

Beyond combination approaches, programmes are increasingly emphasising dose‐shaping—gradual titration, slower exposure ramps or profiles intended to blunt peak‐related symptom intensity while maintaining sufficient receptor engagement for appetite suppression. Although the complex structure of the GDF15–GFRAL–RET complex enforces a steep, effectively binary dose–response relationship regarding receptor recruitment [6, 11], recent pharmacological efforts hypothesise that the *quality* of the downstream signal may nonetheless be adjustable. This concept of ‘biased agonism’ relies on the observation that RET activation engages multiple distinct intracellular cascades, most notably the ERK1/2 and PLC*γ* pathways [7].

Theoretically, next‐generation ligand formats could be engineered to stabilise specific receptor conformations that preferentially favour anorectic signalling pathways while minimising the activation patterns—such as intense PLC*γ* engagement—that are most tightly associated with visceral malaise [23]. However, the translation of this mechanistic nuance remains challenging; the high cooperativity of the signalling complex means that once RET is engaged, the transition to maximal activation is rapid, potentially overriding subtle biases in effector coupling [6]. Consequently, while biased agonism offers a logical route to widen the therapeutic window, it remains to be proven whether the ‘binary’ nature of receptor assembly can be sufficiently decoupled from downstream signal diversification to avoid the aversive responses observed in clinical settings [25].

In contrast to obesity programmes, where the challenge is tolerability, antagonism of GDF15 signalling is being explored for conditions characterised by pathological anorexia and weight loss. In chronic illness states with elevated circulating GDF15, blocking the signal may mitigate sustained hypophagia and support nutritional rehabilitation [5]. Translationally, this direction has distinct endpoints and risks: Benefit should be anchored to function and quality of life (not weight alone), and careful patient selection is required because GDF15 is a stress‐responsive signal that may play adaptive roles in illness behaviour [5, 27].

To contextualise emerging GDF15–GFRAL agonism within the current landscape of obesity management, Table 3 summarises representative agents and their reported outcomes.

**Table 3:** Comparison of emerging GDF15 agonists and incretin‐based pharmacotherapy.

Semaglutide	GLP‐1 receptor agonist	Phase 3 (STEP 1)	~15.0%	GI side effects; plateau in ~30% of patients
Tirzepatide	GIP/GLP‐1 dual agonist	Phase 3 (SURMOUNT‐1)	~22.5%	GI side effects; long‐term cost
LY3463251 (Lilly)	GDF15 receptor agonist	Phase 1 (NCT04144628)	~4.7% (12 weeks)	Significant nausea (> 40% of participants)
MBL949 (Janssen)	GDF15 receptor agonist	Phase 2b (discontinued)	Minimal efficacy	Failed to translate primate efficacy to humans
AMG 171 (Amgen)	GDF15 receptor agonist	Phase 1 (terminated)	N/A	Programme halted in early development
QL1005	GLP‐1/GDF15 dual agonist	Preclinical	~10%–15% (primates)∗	Translation to humans unproven

*Note:* The asterisk ( ^∗^) indicates that the weight loss is derived from preclinical studies in non‐human primates rather than human primate clinical trial.

Overall, early clinical experience supports the biological validity of targeting GDF15–GFRAL, but it also shows why straightforward ‘more agonist’ approaches struggle: Engineered exposure can address half‐life issues, yet durable, clinically meaningful weight loss depends on whether efficacy can be delivered below the aversion threshold. The most credible routes forward are therefore exposure shaping, dose‐sparing combinations (including dual agonists such as QL1005) and next‐generation formats designed to improve benefit–risk rather than simply increase potency [5, 23–28, 32].

## 6. Biomarker Value and Clinical Interpretation

Beyond its role as a therapeutic target, GDF15 has emerged as a nuanced circulating biomarker of metabolic stress. Its utility in obesity medicine lies in its correlation with systemic bioenergetic strain rather than simple adiposity. In observational cohorts, circulating GDF15 concentrations are positively correlated with BMI, insulin resistance and age, creating an apparent paradox in which high levels of an anorectic hormone coexist with progressive obesity [33, 34]. In addition, the sensitivity of the GDF15–GFRAL axis may be altered in obesity, as MT1‐MMP–mediated cleavage of GFRAL can reduce receptor abundance and blunt GDF15 signalling, therefore providing a mechanistic explanation for the apparent GDF15 resistance in chronic metabolic disease [35].

The dynamic nature of this relationship is particularly evident following bariatric surgery, where GDF15 levels rise after Roux‐en‐Y gastric bypass and sleeve gastrectomy. Clinically, the magnitude of this elevation has prognostic value, as studies indicate that higher postoperative GDF15 levels predict greater total weight loss at 12 months, independent of baseline BMI. These findings support the potential utility of GDF15 as a biomarker for identifying physiological responders to bariatric surgery.

Furthermore, GDF15 serves as a critical pharmacodynamic readout for pharmacological interventions, most notably metformin. It is now established that metformin′s weight‐suppressive effects are partially mediated through the GDF15–GFRAL axis. Administration of metformin triggers the ISR in hepatocytes and enterocytes, resulting in a twofold to threefold increase in circulating GDF15 [19, 36]. This introduces an important interpretive consideration for clinicians. In patients with type 2 diabetes and obesity, an elevated baseline GDF15 may indicate ongoing metformin therapy rather than progression of metabolic disease or increased endogenous stress. In this population, elevated GDF15 levels correlate with insulin resistance, hyperglycaemia and type 2 diabetes progression, serving as a biomarker for disease severity [37, 38]. GDF15 enhances insulin sensitivity and glucose tolerance by promoting oxidative metabolism in macrophages and suppressing appetite via GFRAL receptor signalling, potentially aiding weight loss in obesity [38, 39]. However, in diabetes, paradoxically high levels fail to fully mitigate insulin resistance, reflecting compensatory anti‐inflammatory responses [40]. Circulating GDF15 rises with complications like diabetic retinopathy, associating positively with fasting glucose, HbA1c and endothelial dysfunction [41]. It protects vasculature and beta‐cell survival by downregulating NF‐*κ*B pathways, yet sustained elevation signals poor prognosis in obese diabetics [39, 40]. Overall, GDF15 offers therapeutic potential targeting obesity‐driven diabetes pathology.

While the focus remains on metabolic health, accurate interpretation of GDF15 requires acknowledging its pleiotropic effects. Because the peptide is renally cleared, chronic kidney disease—a frequent comorbidity in obesity—can artificially inflate circulating levels. This necessitates adjustment of the glomerular filtration rate (GFR) to avoid false‐positive risk stratification [21].

Marked increases in GDF15 are also seen in physiological states such as pregnancy, particularly in hyperemesis gravidarum, as well as in pathological conditions like heart failure and cancer cachexia [8, 19, 28]. It is essential to recognise these nonmetabolic sources to ensure that elevated GDF15 is correctly attributed to metabolic strain, rather than to unrecognised malignancy or renal impairment.

Integrating GDF15 measurement into obesity management aligns with a precision medicine approach. The principal clinical interpretations and therapeutic implications of GDF15 are summarised in Table 4. Rather than serving as a static marker, GDF15 should be viewed as a dynamic indicator of metabolic strain. In clinical practice, stratifying patients by endogenous GDF15 levels may help identify those with an intact and responsive GDF15–GFRAL pathway who could benefit from receptor agonists, whereas individuals with persistently high levels may represent pathway saturation or resistance and require alternative therapeutic strategies.

**Table 4:** Clinical interpretation and therapeutic relevance of GDF15. An overview of the translational implications of GDF15 biology in obesity, cachexia and pharmacological management.

Context	Interpretation	Relevance	Key references
Obesity	Elevated GDF15 reflects metabolic strain rather than effective satiety.	Explains the ‘GDF15 paradox’ where high levels coexist with obesity.	[19, 30]
Cancer cachexia	Pathological activation of central anorectic signalling by tumour‐derived GDF15.	Provides a rationale for GDF15 antagonism (antibodies) to treat wasting.	[11]
Pharmacological agonism	Human weight loss efficacy is constrained by dose‐limiting nausea.	Clinical translation is hindered by a narrow therapeutic index.	[23, 26]
Metformin therapy	Metformin induces GDF15 via the mitochondrial ISR.	Explains the GDF15‐dependent weight‐loss effects of metformin.	[16, 32]
Combination strategies	Synergistic effects with GLP‐1 receptor agonists.	Dual‐acting molecules may improve weight loss while minimising nausea via dose‐sparing.	[28]

Abbreviations: GDF15, growth differentiation factor 15; GLP‐1, glucagon‐like peptide‐1; ISR, integrated stress response.

## 7. Conclusion

GDF15 is a stress‐responsive endocrine signal that suppresses feeding via a specific brainstem receptor system. The discovery of GFRAL in the AP and NTS has established GDF15 as a defined neuroendocrine axis connecting peripheral cellular stress to adaptive behavioural responses [6, 7].

In the context of obesity treatment, the GDF15 pathway is of interest because it functions independently of classical homeostatic satiety mechanisms, but it also reflects a broader biology of systemic metabolic strain. At the same time, the relevance of this pathway is shaped by its complexity: Initial human studies suggest that pharmacological activation of this axis can reduce appetite and body weight, although tolerability, particularly nausea and aversion, remains a significant limitation to clinical application [28, 30]. Its broad range of effects requires careful phenotyping to distinguish metabolic stress from other causes, such as pregnancy‐related syndromes, malignancy or advanced organ dysfunction [8].

Overall, the GDF15–GFRAL axis represents a promising but complex target in obesity medicine. Its future clinical utility will depend on further mechanistic understanding, improved patient stratification and robust evidence from ongoing clinical studies evaluating whether efficacy can be achieved with a favourable profile.

## Author Contributions

Kamil Ahmed: conceptualization (equal), writing—original draft (equal), review and editing (equal) and approval of the final version submitted for publication. Asia Saturnino: conceptualization (equal), writing—original draft (equal), review and editing (equal) and approval of the final version submitted for publication. Paolo Pozzilli: conceptualization (equal), writing—original draft (equal), review and editing (equal) and approval of the final version submitted for publication.

## Funding

Open access publishing facilitated by Universita Campus Bio‐Medico di Roma, as part of the Wiley ‐ CRUI‐CARE agreement.

## Disclosure

The authors have nothing to report.

## Conflicts of Interest

The authors declare no conflicts of interest.

## Data Availability

The data that support the findings of this study are available from the corresponding author upon reasonable request.
